# Remote Monitoring Systems for Chronic Patients on Home Hemodialysis: Field Test of a Copresence-Enhanced Design

**DOI:** 10.2196/humanfactors.7078

**Published:** 2017-08-29

**Authors:** Na Liu, Jinman Kim, Younhyun Jung, Adani Arisy, Mary Ann Nicdao, Mary Mikaheal, Tanya Baldacchino, Mohamed Khadra, Kamal Sud

**Affiliations:** ^1^ School of Information Technologies Sydney Australia; ^2^ Biomedical and Multimedia Information Technology (BMIT) Group School of Information Technologies Sydney Australia; ^3^ Nepean Telehealth Technology Centre Nepean Hospital Kingswood Sydney Australia; ^4^ Home Haemodialysis Unit Regional Dialysis Centre Blacktown Hospital Sydney Australia; ^5^ Department of Renal Medicine Nepean Hospital Kingswood Sydney Australia; ^6^ Sydney Medical School (Nepean Clinical School) The University of Sydney Sydney Australia

**Keywords:** remote-monitoring, home hemodialysis, co-presence enhancement, design

## Abstract

**Background:**

Patients undertaking long-term and chronic home hemodialysis (HHD) are subject to feelings of isolation and anxiety due to the absence of physical contact with their health care professionals and lack of feedback in regards to their dialysis treatments. Therefore, it is important for these patients to feel the “presence” of the health care professionals remotely while on hemodialysis at home for better compliance with the dialysis regime and to feel connected with health care professionals.

**Objective:**

This study presents an HHD system design for hemodialysis patients with features to enhance patient’s perceived “copresence” with their health care professionals. Various mechanisms to enhance this perception were designed and implemented, including digital logbooks, emotion sharing, and feedback tools. The mechanism in our HHD system aims to address the limitations associated with existing self-monitoring tools for HHD patients.

**Methods:**

A field trial involving 3 nurses and 74 patients was conducted to test the pilot implementation of the copresence design in our HHD system. Mixed method research was conducted to evaluate the system, including surveys, interviews, and analysis of system data.

**Results:**

Patients created 2757 entries of dialysis cases during the period of study. Altogether there were 492 entries submitted with “Very Happy” as the emotional status, 2167 entries with a “Happy” status, 56 entries with a “Neutral” status, 18 entries with an “Unhappy” status, and 24 entries with a “Very unhappy” status. Patients felt assured to share their emotions with health care professionals. Health care professionals were able to prioritize the review of the entries based on the emotional status and also felt assured to see patients’ change in mood. There were 989 entries sent with short notes. Entries with negative emotions had a higher percentage of supplementary notes entered compared to the entries with positive and neutral emotions. The qualitative data further showed that the HHD system was able to improve patients’ feelings of being connected with their health care professionals and thus enhance their self-care on HHD. The health care professionals felt better assured with patients’ status with the use of the system and reported improved productivity and satisfaction with the copresence enhancement mechanism. The survey on the system usability indicated a high level of satisfaction among patients and nurses.

**Conclusions:**

The copresence enhancement design complements the conventional use of a digitized HHD logbook and will further benefit the design of future telehealth systems.

## Introduction

End stage renal disease (ESRD) is the most severe form of chronic kidney disease (CKD), and patients suffering from this condition have poor life expectancy if left untreated. Patients who are not suitable for a transplant have to remain on dialysis for the rest of their lives, making dialysis an essential life-prolonging treatment modality for patients with ESRD [1]. Dialysis replaces kidney function through the removal of accumulated metabolic waste products, by a process of diffusion, as well as removal of excess fluids from the body, by a process of ultrafiltration [2]. Dialysis can essentially be performed by two modalities: peritoneal dialysis, which uses the patients’ own peritoneal membrane, and hemodialysis, which uses a synthetic membrane for diffusion and ultrafiltration to occur. While chronic peritoneal dialysis is usually performed by patients at home, hemodialysis is typically conducted for 4-5 hours 3 times a week in a hospital setting (in-center hemodialysis) or in a community setting (satellite hemodialysis). In addition, patients (or their families) can also be trained to conduct hemodialysis treatments at home (home hemodialysis).

Home hemodialysis (HHD) has a number of advantages over other forms of dialyses as it leads to better patient survival, better quality of life, greater independence and opportunity for rehabilitation [3], and is more cost effective [4]. However, HHD is not without its drawbacks. Although patients and their families are trained to deliver these seemingly complex treatments at home, patients on HHD often feel abandoned by the health system because of a constant lack of real-time oversight by trained health professionals, which may increase patients’ anxiety [5]. This lack of oversight may also promote noncompliance, such as violating dietary and fluid intake restrictions [6,7], noncompliance to medications [6], and skipping or shortening dialysis sessions [8]. Moreover, the feelings of isolation and difficulty in accessing assistance may also lead to lack of confidence and poor decisions, including abandoning this otherwise very useful and effective dialysis modality [9] and increasing the risk of complications [6]. The common practice to address some of the limitations associated with HHD is to make frequent phone calls or home visits and use paper-based logbooks to record a number of dialysis-related parameters of hemodialysis treatments conducted at home. However, these paper-based logs can be reviewed only when a patient sees their health care professional face-to-face. The problem with this approach is that it could take a long time until the patient sees their health care professionals, rendering it impossible for them to take early corrective actions for any worrisome deviations in these parameters. Additionally, patients may forget to bring their logbooks at the time of consultations or logs could be lost or unintentionally erased before their health care professionals have a chance to review them.

Although there is no system specifically designed for HHD, there are multiple attempts at addressing the identified limitations above for other health conditions. There are a number of mobile phone apps developed to be used as simple self-monitoring or logging apps. Medical professionals are also devoting efforts to building customized computer-based self-monitoring systems [10,11], with features such as summative information of health signs, and electronic reminders sent at a predefined frequency. Some of those apps are similar to paper-based logs, while others may have additional functions to remind patients to report their vital signs in a timely manner. Although with the setting of reminders, noncompliance might be improved. However, the lack of presence of health care professionals onsite (ie, at home for patients on HHD) may still make patients feel isolated and anxious about whether their dialysis-related parameters are stable and within expected range and anxious about whether treatments are being monitored by trained health professionals. Prior study has pointed out that addressing social isolation and emotional needs of users is a major challenge to the emerging telemonitoring and smart care technologies [12].

In this study, we propose an exemplar design for an HHD system optimized for HHD patients with novel mechanisms to enhance patient’s perceived “copresence” with their health care professionals. Our design addresses the social and emotional needs of the patients. The pilot deployment of the HHD system employs multimethod data collection including system entries, survey questionnaire, and interview. The study aims to reveal how patients perceive and utilize the functions related to emotion sharing and copresence enhancement. The results will demonstrate how the feelings of being connected with their health care professional can be improved to enhance patients’ experience on HHD. This study presents the system design and the analysis of the impact of the copresence enhancement mechanism. The clinical improvements from the pilot, such as change in dialysis prescription, patient and staff time-saving associated with consultation and travel times, and user satisfaction, were presented as a separate study [13]. The rest of the paper is organized as follows: related work on information technology (IT)‒enabled self-monitoring is reviewed, followed by a discussion of theoretical foundation informing the design. System development will then be discussed, followed by system usability evaluation.

### Background

IT-enabled patient monitoring systems play an important role in well-being and chronic illness management. They are changing the way health services, patient data, and medical interferences interact and are able to reduce the number of hospitalized patients, minimize the load on clinical staff, and lower the total caring costs for governments. In general, IT-enabled patient monitoring systems would benefit both patients and medical professionals by providing digitization of and rapid access to health information. They have been used to monitor various types of illness, including cardiac and heart illness [14-16], diabetes [14,17,18], mental illness [19,20], asthma [21], obesity [22], and other types of illness.

Prior studies on patient monitoring systems usually consist of three main components: (1) tracking physiological parameters, such as respiration rate, heart rate [23], blood pressure, and blood glucose level [24], some of which are able to be captured by wearable sensors [25] while others rely on patients’ self-input [26]; (2) a dashboard for clinicians to view data through a Web interface or mobile interface enables authorized personnel to monitor the patients’ condition and facilitate remote diagnosis; and (3) a messaging function to provide reminders or alerts to both patients and physicians. These systems are continuously being enhanced, but challenges remain to improve their clinical impact. Data security and privacy are believed to be major threats to IT-enabled patient monitoring systems, especially in terms of patient identification and confidentiality of medical information [27]. Another concern is the battery life or energy consumption of the design, as continuous data collection and processing can impose on a phone’s battery runtime [27].

Prior research suggests that addressing social isolation and emotional needs of users is a challenge to the emerging telemonitoring and smart care technologies [12]. The feeling of isolation is also a serious problem in patients on HHD [5] as there is a lack of face-to-face communications between patients and health care professionals. As the HHD procedures require patients to perform the complex dialysis treatments autonomously, the feeling of isolation from health care professionals may cause anxiety and lower their mood and self-confidence. When patients feel disconnected from their health care professionals, their compliance to medical advice drops and their confidence of self-care also comes down [5]. Thus, in this study we have paid attention to the social nature of using technology and introduce the concept of copresence. We discuss how we designed a remote-monitoring system for patients on HHD to reduce patients’ feelings of isolation.

Copresence, referring to the sense of connection with another interactant [28], exists when people feel that they are actively perceiving others and feel that others are also actively perceiving them [29]. Specifically, copresence refers to the perception by a communicator that another person in a mediated or online environment is real, immediate, or present [30]. Thus, copresence is a reflection of psychological connection to and with another person. It is required that interactants feel they were able to perceive their interaction with a partner and that their interaction partner actively perceived them [28].

Copresence was widely studied in the field of human-computer interactions, and its application has been used in the context of virtual team collaboration [31] and online shopping experiences [32]. Higher perceived copresence directly influences the satisfaction of the communication medium [28]. However, existing studies of remote patient monitoring have not yet capitalized on the importance and capabilities of copresence.

## Methods

A field trial involving 3 nurses and 74 participants was conducted to evaluate the copresence-enhanced HHD system, over a 6-month period.

### The Home Hemodialysis System Architecture

**Figure 1:**
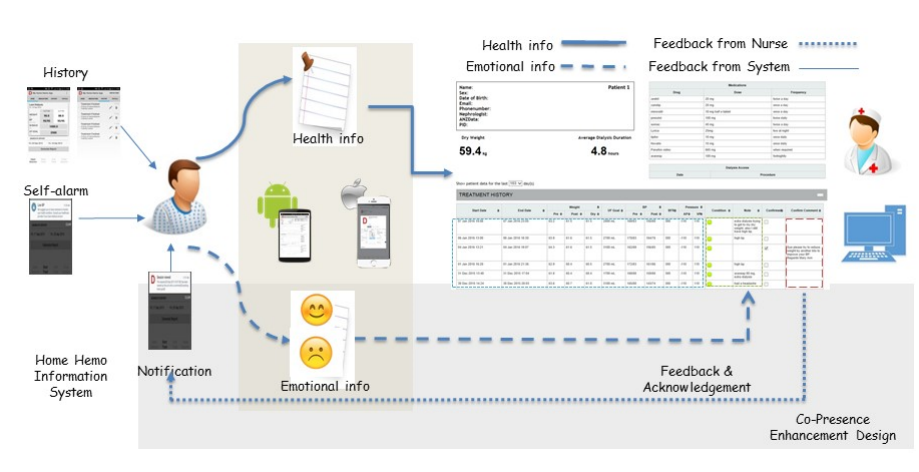
Design of the copresence-enhanced home hemodialysis system.

The home-monitoring system consisted of three main components. The first component is the HHD app installed in the patient’s mobile device. The second component is the cloud server built on Windows Azure services with SQL storage as the database. The third component is the Web app also hosted on the Azure server. Azure provides industry-leading protection and privacy of the data. Patients use their own mobile device to record their hemodialysis-related data. These data are sent immediately whenever the mobile device has an Internet connection. After the Azure cloud service retrieves the data, it is stored in its database. Patients’ health care professionals (including their HHD nurses and renal specialists) can choose to access the website at any time. The website provides the patient’s up-to-date status with regards to their HHD treatment parameters, along with trends in these parameters over time, enabling the clinical team to make appropriate decisions on a patient’s dialysis prescription, blood pressure, and body weight that are a surrogate of their body fluid status. Reports along with trends in various parameters over time can be generated and saved as printable documents. Figure 1 illustrates the described functionality, users, and the exchange of information and emotion between the patients and the health care professionals. The detailed functions of the HHD system and the copresence enhancement features will be described below.

### The Basic Functions

The basic functions of the self-monitoring system allow patients to record their hemodialysis session data (eg, pre- and postdialysis body weights, blood pressures, ultra-filtration volumes, blood flow rates, venous and arterial pressures, session times) to the system and get alerts on abnormal parameters or if they did not perform dialysis within a certain amount of time. At the end of each dialysis session, patients report their status on how they are feeling on a 5-point sliding scale, to flag if future dialysis sessions may need to be modified. Patient are also able to enter shorts notes in the textbox of the app for each entry submission. Health care professionals can review patient data, either in real time or at intervals based on patients’ clinical needs, thereby allowing monitoring of patients’ parameters as well as noncompliance to dialysis regimens, skipping dialysis sessions, or shortened dialysis sessions. They can also get a list of patients who have an abnormal status at the end of hemodialysis sessions, prompting remote analysis of their dialysis data to take remedial actions for their subsequent hemodialysis treatments.

### The Copresence Enhancement Mechanisms

**Figure 2:**
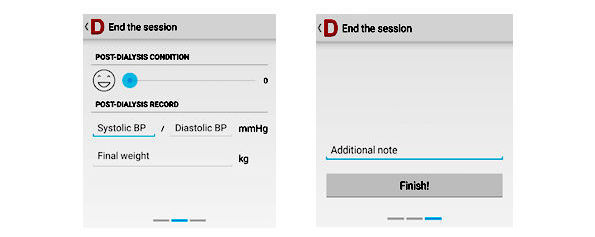
Patients’ interface to input their emotions.

**Figure 3:**
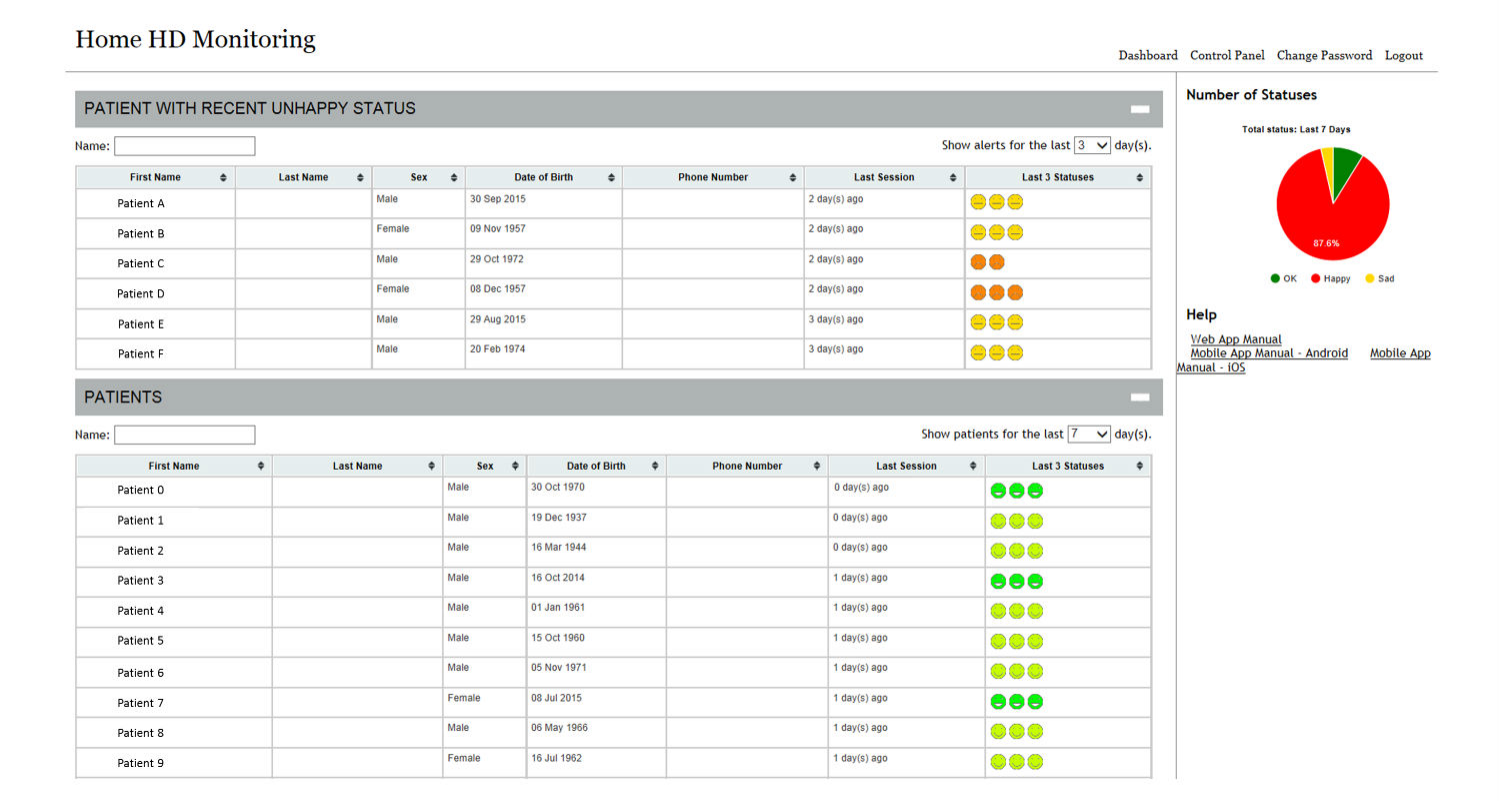
Dashboard view of patients’ emotions.

Besides the basic functions that provide the digital data log and enable remote monitoring of patients, copresence enhancement mechanisms are related to the feeling of connection between two people. Given its dual nature, this usually consists of two perspectives, including a participant’s perception of their partner’s involvement in the interaction (perceived others’ copresence) and a participant’s own involvement in the interaction (self-reported copresence) [33]. The realization of copresence requires mutual synchronization attention and emotion in a computer-mediated environment [34]. Our system enables patients to rate their emotions at the end of the dialysis session as part of the self-health reporting exercise, so that health care professionals can have a general understanding of patients’ feelings at the end of their dialysis sessions. The emotions are reported on a 5-point scale, with 1=Very Happy, 2=Happy, 3=Neutral, 4=Unhappy, and 5=Very Unhappy. Figure 2 shows the interface in the app that allows patients to share their emotions. Patients are also allowed to include text as additional comments to each submission (Figure 2). Figure 3 provides an interface of the dashboard on how the emotions are reviewed from the health care professionals’ side. Dialysis data of patients expressing a low mood are reviewed as a priority.

**Figure 4:**
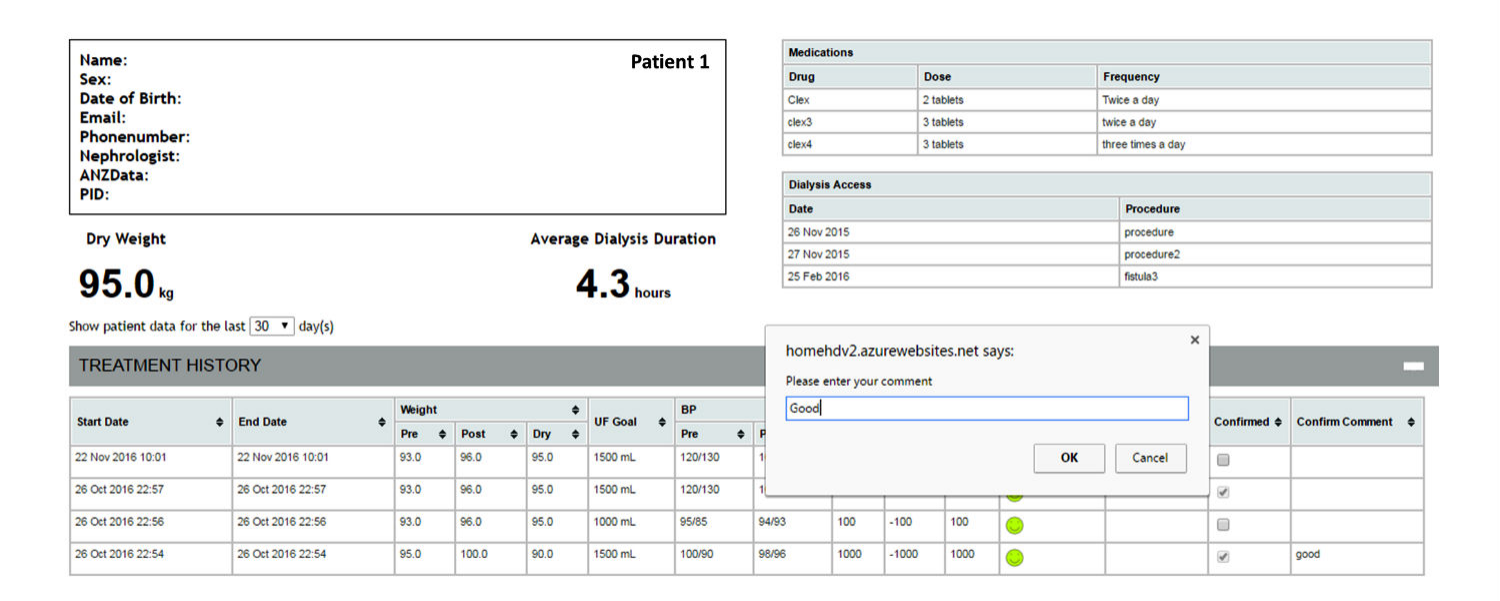
Sending feedback to patients.

**Figure 5:**
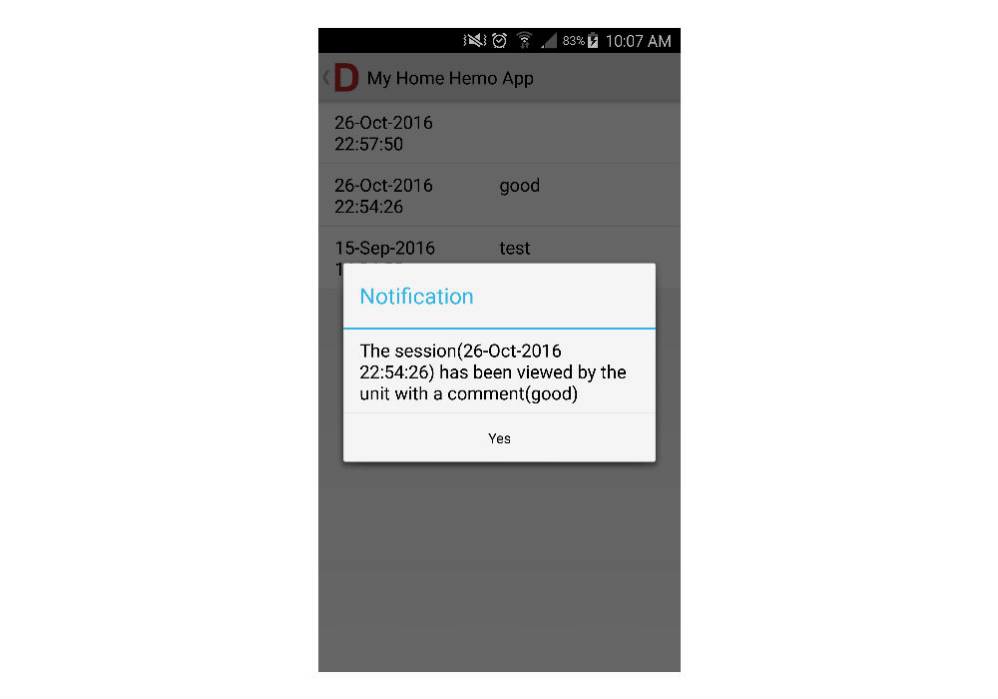
Patients’ view of health professional’s feedback.

Health care professionals can also send feedback (with or without comments) by simply clicking the confirmed function in the system, to let patients know their dialysis data have been reviewed (Figures 4 and 5).

The features of sharing emotions and one-click feedback functions were designed to collectively enhance the mutual attention and emotion between patients and health care professionals, while not increasing staff workload significantly.

### Timeline of the Study and Data Collection

In order to evaluate the effectiveness of the system in general and the copresence enhancement mechanisms, a field trail was conducted with users at the Home Hemodialysis Unit at the Regional Dialysis Unit, Blacktown Hospital in Sydney. The Home Hemodialysis Unit is part of the Western Renal Service in Western Sydney that has a philosophy of promoting home dialysis and is one of the largest home dialysis services in Australia.

**Figure 6:**
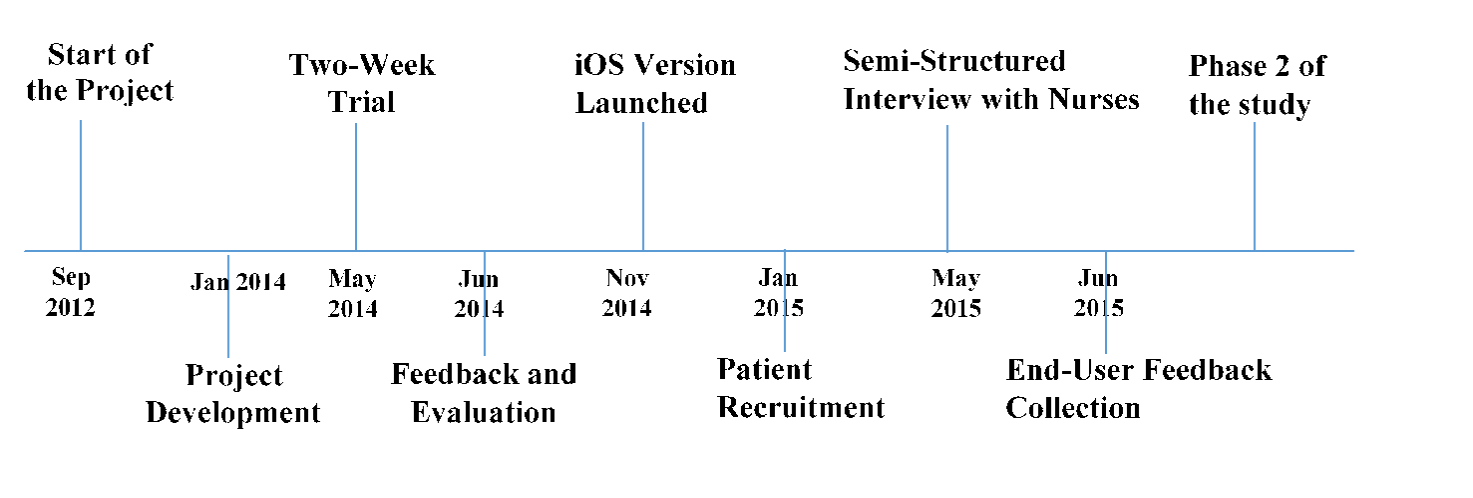
Timeline of the study.

A 2-week trial to test the app’s functionality on an Android platform commenced initially with 10 patients and 2 health care professionals (nurses). Improvements were made based on the feedback received, and an iOS version was launched along with updated Android version. Patient recruitment was scaled up to 74 patients and also to 3 nurses. The timeline of the study is summarized in Figure 6.

Once the HHD system was implemented within the Unit, an audit was conducted wherein qualitative data were collected through semistructured interviews to understand the efficacy of the copresence enhancement mechanism with nurses and patients. The interview was structured based on the system evaluation: ease of use, reliability and performance, and usefulness. The patients were allowed to give general comments along these three dimensions. They were further probed to explain whether they felt better connected with the health care professionals and whether they were more confident in doing their dialysis at home. Content analysis techniques were used for analyzing the qualitative data obtained. Content analysis is a research tool used to determine the presence of certain words or phrases within texts, and from these, infer the meanings that underlie these passages of text [35]. Researchers use these techniques to make inferences about the messages within the texts by analyzing the presence, meanings, and relationships of certain words and concepts [35]. The purpose is to reveal the insights related to the system usage rather than establishing casual relationships.

Basic quality assurance surveys were also conducted with the patients, where patients were required to answer a few questions in a 5-point Likert form to evaluate the systems along three dimensions: ease of use, reliability and performance, and usefulness. The questions were adopted from prior validated instruments with minor modification to the context of dialysis patients. Sample survey questions and interview protocols are included in Multimedia Appendix 1. The interview data were analyzed together with basic quality assurance survey to look at patients’ and nurses’ feedback on general system usage, copresence enhancement mechanisms, and effectiveness of the system. System data including the frequency of self-reporting and the time being acknowledged are also analyzed.

## Results

### General Information

**Figure 7:**
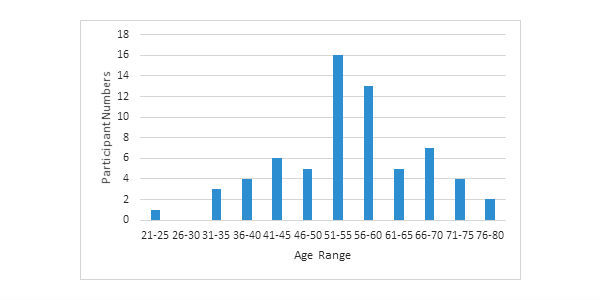
Participants' age distribution.

Among the 74 participants of the study, there were 25 female and 49 male patients. The age distribution is shown in Figure 7 with more than half of the patients over 50 years old. The oldest patient was 78 years old and the youngest 21 years old.

There were altogether 2757 entries created by the patients during the trial period (Table 1). The average duration for the patients participating in the study was 128 days, with a standard deviation of 46.5 days. The minimum duration of participation is 9 days while the maximum is 180 days. Based on the results of the quality assurance survey and semistructured interview, both patients and nurses reported high ease of use and usefulness of the system. The results were further analyzed by patients and staff.

### Analysis of Patients

Frequency and duration of using the app was dependant on the date the patient was enrolled in the trial. The total number of entries reported with a different emotional status is summarized in Table 1. Altogether there were 492 entries submitted by indicating “Very Happy” as the emotional status, 2167 entries with a “Happy” status, 56 entries with a “Neutral” status, 18 entries with an “Unhappy” status, and 24 entries with a “Very unhappy” status. On average, each patient had 37 entries for hemodialysis cases during the period of pilot study, with a standard deviation of 27.8. The maximum number of entries created by patients is 91, and the patient duration of participation is 180 days.

**Table 1:** The number of entries patients made during the evaluation period.

	Entries	Very happy	Happy	Neutral	Unhappy	Very unhappy
Entries, total N	2757	492	2167	56	18	24
Percentage of different emotional status, %	100	17.8	78.6	2.03	0.65	0.87
Average entries per patient, n	37	6.6	29.3	0.76	0.24	0.32

Patients reported an average of 4.2 on ease of use of the system (SD 0.77). Patients also reported an average of 4.1 for reliability and performance of the system (SD 0.87). The average perceived usefulness of the system was 4.1 (SD 1.4). They also gave positive feedback regarding the usage of the system during their interviews.

The patients also showed greater awareness of copresence enhancement design mechanisms that we associate with the interview responses such as “not feeling alone,” “knowing I am monitored,” and other similar phrases. They reported that they felt relaxed to know that their dialysis parameters and treatments were being monitored, especially when they received acknowledgements from the nurses after submitting their dialysis-related data. When asked about the functions of entering notes and emotions into the system, one of the patients mentioned, “It is comforting to know that my results are being monitored and you are not alone.” As the negative modes (Very unhappy and Unhappy) usually get the fastest response from the health care professionals, the patients who expressed negative emotions also expressed that they felt “care and responsiveness from the health care professionals all the time.” The copresence enhancement design of the system was shown to improve the mutual attention and mutual emotion for patient-nurse communication. The patients mentioned being “motivated to keep dialysis schedule” by the features in the app. In addition, the patients mentioned that they may append some notes to some submitted entries as they know “the nurse will get the message.”

We further analyzed the entries with notes appended. Out of the 2757 entries created during the study period, 989 entries were submitted with short notes. We compared the notes entered against the emotion status submitted (see Table 2). Among 492 entries with the “Very Happy” status, 256 entries were supplemented with patients’ short notes. For the “Happy” status, 30.69% (665/2167) of entries had additional notes. For “Neutral” status, 59% (33/56) had text notes in the entries. For the “Unhappy” status, all 18 entries consisted of notes input from the patients. For the “Very unhappy” status, 71% (17/24) of entries had notes input. The result shows that when patients are in negative mood, they would like to express themselves with the support of text messages and to improve nurses’ understanding on their status. The patients also expressed that: “When I am not feeling well, I want to get the nurses’ immediate attention” and “I am glad that they are paying attentions to our problems and emotions.”

We also coded the notes entered by the patients into the system, which fell into three categories: (1) describing health status, such as “a bit dizzy in the last hour,” “notice my BP increasing,” “my bleeding didn’t stop quickly it took almost 2 hrs,” “all good,” (2) greeting to health care professionals, such as “thanks, Maryann,” “have a nice day,” “A big smile”; this type of message is very common for the entries “Very happy” and “Happy” emotions, and (3) expressing technical difficulty, such as “Power failure after 30 mins.” The patients mentioned that that by entering notes to the system, their queries can be quickly attended by the health care professionals and they do not need to wait till next consultation. They also mention it is an effective way to communicate with staff and feel they are “virtually connected” with the staff all the time.

**Table 2:** The number of entries submitted with text notes.

	Notes	Entries with notes for “Very Happy”	Entries with notes for “Happy”	Entries with notes for “Neutral”	Entries with notes for “Unhappy”	Entries with notes for “Very Unhappy”
Total N	989	256	665	33	18	17
Percentage by type of emotions, %	100	25.9	67.2	3.3	1.8	1.7
Average per patient, n	13	3.5	9.0	0.45	0.24	0.23
Note average per emotion entry, %		52	31	59	100	71

### Analysis of Clinical Staff

With the implemented system, there were on average 12 patients remotely reviewed per week. It resulted in savings of 7 hours in nursing and patient times each, from reduced home and/or unit visits, equating to a total saving of 11 hours of travel time and 544 kilometres of travel distance. This paper focuses on the copresence mechanism; additional details about the operational benefits can be found in another paper [13]. The nurses reported satisfaction with the system features during the interview, especially by mentioning it is a “time saving tool.”

The nurses mentioned that getting a glimpse of patients’ emotional status with a simplified rating was reassuring. They reported a positive feeling of being able to “reach out” to more patients. The notes entered by the patients also provide a better understanding of whether the problem was technical or illness-related. When seeing some greeting messages, the nurse also said that these patients were also treating them as friends, so the system still maintains a certain level of connection between patients and them. The digital log kept a history of patients’ dialysis-related data and allowed nurses to track changes in the patient’s dialysis parameters over time. The system also enabled nurses to change dialysis prescriptions and patients’ dry weights in a timely manner, prompting patients to know that they were continuously monitored by nurses.

## Discussion

### Principal Considerations

This study is one of the first studies on copresence in remote monitoring in health care settings. Despite many efforts devoted to building and understanding the effectiveness of computer-based self-monitoring systems [10,11], few studies have looked at the importance of enhancing the social emotional needs of patients. Our study indicates that equipped with the copresence enhancement mechanisms, the HHD system received positive feedback from both patients and nurses. The field trial implies that HHD might reduce patients’ feeling of isolation and anxiety caused by independently conducting hemodialysis treatments at home.

Although the system usage was voluntary for the patients and they could still enter their HHD-related data into the conventional exercise books without using the app, this mixed method study revealed that the app usage has achieved its effectiveness as designed. With the feeling of being monitored and connected to their nurses at all times, patients’ motivations to adherence was increased. The system empowered patients to better understand and take care of their health care and therefore suggests that the system has the potential to improve patient uptake and retention on HHD programs and improve relationships with their nursing staff. The nurses also felt assured to have a simplified view of patients’ emotions and dialysis parameters.

Our study has demonstrated the effectiveness of copresence enhancement mechanism in the context of remote monitoring of dialysis parameters in patients on HHD. IT-enabled patient monitoring is a trend and the benefits have been demonstrated including reducing nurse-patient ratio, reducing operational cost, and improving data accuracy. There are also issues and challenges with the use of technology, and a significant one is that patients are not able to have face-to-face communication with health care professionals and may feel isolated and lose compliance and confidence through the self-disease management process. While current ways to reduce patient isolation require additional investment of physicians’ and nurses’ time, such as increasing standby hours or having a video conferencing call, our study proposes a concise design by introducing functions like sharing emotions using emojis, sending quick notes, sending acknowledgment, and having a prioritized response to patients’ records. The features provide health care professionals a preliminary filtering of patients’ situations and provide patients the feeling that they are cared for and monitored constantly.

Our study also highlighted the importance of communicating emotions with health care professionals. Prior studies on telecare suggest that social and emotional needs of users are overlooked in current solutions [12]. Patients expressed that better connections were built with staff through submitting emotional feedback for the dialysis session. The design of one-click feedback function is also highly regarded by the nurses.

### Limitations and Future Work

This study has a few limitations, but it opens up exciting avenues for future research. First, there is no comparison group in the study, since the aim was not to establish causal relationship, but rather to reveal in-depth insights based on a combination of subjective and objective data. Future study can plan for a randomized controlled trial and draw causal relationships between system usage and clinical benefits. In the future, we plan to conduct a prospective study to measure the long-term benefits to patients and on the efficacy and productivity of health care professional’s care delivery with the use of our copresence enhancement mechanisms. Second, feelings such as isolation were not directly measured using questionnaires. The current study only interpreted such meanings based on the qualitative data collected through interview. Future studies should include quantitative measurements of feelings and emotions. Third, there might be other possible features that can be implemented to enhance copresence. For example, studies have found that using an avatar can enhance perceived copresence in the context of teleconferencing and virtual learning [36]. As this study was to test the idea of copresence enhancement mechanisms while not overloading patients with too many functions, these features were not implemented in the HHD system. Future study can definitely implement and test the effectiveness of different copresence enhancement features. Additionally, we plan to further complement our copresence mechanism by incorporating a video conferencing tool to improve communication between patients and health care professionals and to ensure the safety of patients doing dialysis at home, as videoconferencing can enable assessment of patients’ fluid status, visual inspection of vascular access integrity, dialysis machine malfunctions, and alarms.

### Conclusion

In this paper, we propose an exemplary design of an HHD system by incorporating copresence enhancement mechanisms. The design was able to address the challenges of monitoring patients’ dialysis-related parameters while they are on HHD and their feeling of isolation when conducting these treatments. Our user-centered HHD system was designed to enhance nurse-patient mutual attention and emotion without overloading them with complicated functions. The proposed mechanisms were shown to improve the feeling of connectedness with clinicians for the patients, improve adherence to their dialysis treatments and schedules, and also enhance emotional well-being of patients. It is one of the first studies to address social concerns and emotional feelings for patients on home dialysis. Our results from patients and the health care team have been positive and affirm that the proposed copresence-enhanced mechanisms have many benefits to HHD. We suggest that our copresence enhancement mechanisms are relevant to other remote chronic disease management systems.

## Appendix

Multimedia Appendix 1 Survey and interview instruments.

## Abbreviations

CKD: chronic kidney disease
ESRD: end stage renal disease
HHD: home hemodialysis
