# Supplementary material for: Remote Monitoring Systems for Chronic Patients on Home Hemodialysis: Field Test of a Copresence-Enhanced Design
Source: JMIR Hum Factors. 2017 Aug 29;4(3):e21. doi: 10.2196/humanfactors.7078 (PMC5596297; doi:10.2196/humanfactors.7078)
Supplement: Multimedia Appendix 1 [file humanfactors_v4i3e21_app1.pdf]

Multimedia Appendix 1. Survey and Interview Instruments

Ease of Use (1 = Strongly Disagree; 5 = Strongly Agree).

- I can easily learn how to use the application.
- I think the navigation in the application is confusing.
- The text in the application is every easy to read.
- This application is easier to use compared to paper-based logbook.
- I imagine most people would learn to use this application very quickly.
- I think that I would need the support of a techical person to be able to use this application.

Reliability and Performance (1 = Strongly Disagree; 5 = Strongly Agree)

- The application takes too long to launch.
- The application takes too long to navigate from one screen to another.
- The application quickly shows my data when I request it.
- The application always shows the correct data.
- When the application alerted me, a proper reason is given.

Usefulness (1 = Strongly Disagree; 5 = Strongly Agree).

- I find it very useful to be able to see my treatment data as a chart.
- I find the alerting feature very useful.
- I think it is very useful that the medical staff (eg, nurse) is able to see my treatment data immediately.
- I would love to keep using this system after the trial is finished.

Semistructured Interview Protocol

1. Overall, are you satisfied with the application?
2. Can you please comment on the questions listed for ease of use?
3. Can you please comment on the questions related to reliability and performance?
4. Can you please comment on the questions listed for usefulnesss? Why? / Which part?
5. What feature do you want to see added into the application?
